# Gut Dysbiosis, Sex, and Vascular Smooth Muscle Cells in Abdominal Aortic Aneurysms

**DOI:** 10.1007/s11883-026-01461-9

**Published:** 2026-09-16

**Authors:** Grant Hatcher, Matthew Kazaleh, Ricky Patil, Gorav Ailawadi, Morgan Salmon

**Affiliations:** 1 Department of Cardiac Surgery, Michigan Medicine, University of Michigan, 2800 Plymouth Road, Ann Arbor, MI 48109 USA; 2 Frankel Cardiovascular Center, University of Michigan School of Medicine, Ann Arbor, MI USA

**Keywords:** Aneurysms, Gut microbiome, Vascular smooth muscle cells, Inflammation, Gender

## Abstract

**Purpose of Review:**

This paper reviews the present literature on how sex-related differences in gut dysbiosis influence vascular smooth muscle cell behavior in the pathogenesis of abdominal aortic aneurysms.

**Recent Findings:**

Emerging research has linked gut microbial metabolites, such as trimethylamine oxide and phenylacetyl glutamine, to abdominal aortic aneurysm pathogenesis via inflammation and vascular damage. Sex hormones also directly influence vascular smooth muscle cell phenotype switching, as estrogen has been found to be protective, while testosterone may enhance inflammation. Novel biomarkers and molecular regulators have emerged as potential targets, revealing new avenues for abdominal aortic aneurysm treatment.

**Summary:**

Gut dysbiosis contributes to abdominal aortic aneurysm progression through inflammation, vascular smooth muscle cell dysfunction, and immune activation. These effects vary by sex and are influenced by sex hormones and structural differences in vascular smooth muscle cells but require further investigation to establish a clear relationship.

## Introduction

Abdominal aortic aneurysms (AAAs) are localized dilations of the sub-diaphragmatic aorta due to compromised wall integrity, defined as greater than 50% dilation or greater than 3 cm in diameter[1]. Most AAAs are asymptomatic and are found incidentally via routine ultrasound screening[2]. AAAs are a significant cause of mortality worldwide, contributing to an estimated 170,000 deaths each year[3]. Epidemiologically, AAA is more prevalent among elderly males; however, female patients demonstrate a higher risk of rupture[4, 5]. Survival following AAA rupture is poor, with mortality approaching 80%, highlighting the need for appropriate identification and intervention[1]. AAAs have been associated with a range of risk factors, including age, male sex, smoking, hypertension, atherosclerosis, and family history. On average, men aged over 65 years are 3–4 times more likely to develop an AAA than women at the same age, but women with AAA have a higher mortality following surgical intervention [6–10]. Additionally, women display faster aneurysm dilation than men following diagnosis and rupture at smaller aortic diameters[7]. Although risk factors have been identified, the underlying biological mechanisms promoting aneurysm degeneration remain obscure. Importantly, the only treatment options available for patients with AAA are open surgery or endovascular aneurysm stent exclusion. Procedural intervention is pursued when the risk of rupture outweighs the surgical risk, which is substantially elevated in patients with cardiac comorbidities, renal failure, or advanced age [3]. Perioperative risks, including mortality, cardiac events, and renal complications, do not outweigh the benefits in patients with small or slowly growing AAAs, for whom surveillance is preferred [9, 11]. Current medical management is limited to risk factor modification, as there are no effective targeted therapies to halt aneurysm growth [12]. This absence of medical options reflects significant gaps in our understanding of AAA pathogenesis and underscores an urgent need for novel therapeutic strategies. Within the aorta, vascular smooth muscle cells (VSMCs) are the principal cell type in the tunica media. VSMCs are essential for maintaining aortic structure and tone, ensuring biomechanical integrity and hemodynamic regulation of pulsatile arterial blood flow [13]. Turbulent flow stimulates oxidative stress, presenting as persistent oxidative signaling and redox-sensitive gene expression, leading to endothelial dysfunction and subsequent changes in vasculature [14]. The need to better understand and target these processes has incentivized further research [15]. The synthetic VSMC phenotype expresses fewer contractile proteins and secretes matrix metalloproteinases (MMPs), weakening the aortic wall. This alteration is advantageous for vascular repair, but a sustained synthetic VSMC phenotype predisposes vessels to AAA and other cardiovascular diseases (CVDs).

The gut microbiome has recently emerged as a potential target for AAA intervention, as it is a regulator of systemic inflammation and vascular health [16]. Emerging studies show significant alterations of gut microbiota, or gut dysbiosis, among AAA patients, indicating a potential contributory role of the gut microbiome in AAA development and progression [16]. Inflammatory metabolites derived from gut microbiota, such as trimethylamine oxide (TMAO) and phenylacetyl glutamine (PAG), have been identified in AAA patients and are implicated in modulating vascular inflammation, endothelial dysfunction, and VSMC behavior upon entering systemic circulation [16]. Notably, these metabolites appear to exert sex-specific effects, possibly contributing to the observed sex differences in AAA incidence and outcomes [17]. This paper aims to integrate current knowledge on the role of VSMC in the AAA pathogenesis with emerging insights into the upstream effects of gut dysbiosis and sex-specific mechanisms. Through examination of these factors that converge on VSMC biology, we hope to identify critical gaps in the literature and provide potential avenues for targeted, therapeutic options for AAA. This topic is especially relevant given the high mortality associated with AAA rupture and the current lack of effective, noninvasive treatment options to prevent aneurysm progression.

### VSMC Dysfunction in AAA

Under physiological conditions, VSMCs maintain a differentiated, contractile phenotype that is essential for vascular tone and structural integrity. This phenotype is characterized by high expression of smooth muscle contractile proteins such as smooth muscle alpha-actin (SMA-α) and calponin and supports extracellular matrix (ECM) homeostasis by producing structural proteins like elastin and collagen, preserving aortic wall tensile strength and elasticity [18–20]. VSMCs also play a key role in regulating vessel diameter and blood flow distribution via Ca^2+^-dependent contraction. This function is modulated by the VSMC microenvironment, including endothelial-derived substances, autonomic nervous system inputs, adrenal hormones, vasoactive peptides, and reactive oxygen species (ROS) [21–24]. However, the contractile phenotype is not fixed; it is regulated in response to environmental, metabolic, and pathological cues [25].

### VSMC Phenotype Switching and Plasticity

VSMCs exhibit remarkable phenotypic plasticity, allowing them to respond dynamically to environmental and pathological stimuli. While this adaptability is essential for vascular repair, it becomes detrimental when chronically sustained, as VSMCs shift from their contractile phenotype to dedifferentiated states that contribute to aneurysm pathogenesis. VSMCs cultured in vitro with elevated lactic acid levels exhibited increased proliferation and migration, along with downregulation of contractile markers, upregulation of synthetic markers, and abnormal morphological changes [26]. Similarly, elevated homoarginine levels promote a transition to an osteogenic phenotype and contribute to vascular calcification, particularly under hyperphosphatemic conditions [24]. These findings indicate that metabolic alterations directly influence VSMC behavior and play a functional role in vascular remodeling.

A hallmark of AAA progression is a phenotypic switch of VSMCs from a contractile to a dedifferentiated synthetic phenotype. This is triggered by pathological stress, such as cytokine exposure, oxidative stress, ECM degradation, or biomechanical strain. When VSMCs undergo this phenotypic switch, they stop producing their contractile markers and take on a phenotype that is highly proliferative, migratory, and secretory [11]. Dedifferentiated, synthetic VSMCs secrete pro-inflammatory cytokines (e.g., IL-1, IL-6 and TNF-α), chemokines, and MMPs that support immune cell recruitment, vascular inflammation, and medial wall degradation [27–29]. Elastin fragments and other ECM breakdown products act as inflammatory signals called damage-associated molecular patterns (DAMPs), further activating toll-like receptor (TLR) signaling pathways that amplify vascular inflammation and matrix degradation [30, 31]. This phenotype is advantageous for vascular remodeling following vascular injury. In the acute setting, the synthetic VSMC phenotype is better suited to migrate to areas of acute vascular injury to repopulate denuded areas of the arterial wall [32]. While phenotypic modulation is a key adaptive response to mechanical or inflammatory injury, chronic activation promotes persistent inflammation and excessive ECM degradation, thus resulting in aneurysmal degeneration.

In addition to the synthetic phenotype, other de-differentiated phenotypes have also been identified, such as the macrophage-like and osteoblast-like phenotypes [33]. The macrophage-like phenotype is pro-atherogenic; VSMCs with this phenotype exhibit reduced lysosomal acid lipase activity, impairing lipoprotein degradation and increasing their propensity to become foam cells [34]. The osteoblast-like phenotype is marked by the formation of calcifying vesicles, decreased expression of mineralization inhibitors, and deposition of matrix prone to calcification [33]. Factors such as decreased calcification inhibitor concentration, mitochondrial dysfunction, and ER stress influence the transition to this phenotype and promote vascular calcification [33]. Together, these findings emphasize the central role of environmental cues in driving VSMC phenotypic plasticity and underscore the importance of regulatory mechanisms that maintain homeostatic gene expression.

### Molecular Influence on VSMC Phenotype

The phenotypic plasticity of VSMCs is governed by an intricate network of transcriptional and epigenetic regulators that interpret environmental cues and mediate gene expression. Transcription factors such as Kruppel-Like factor 4 (KLF4), myocardin, and transcription factor EB (TFEB) have been linked to the regulation of VSMC phenotypes by controlling gene expression that determines VSMC phenotype. KLF4 suppresses the expression of VSMC contractile marker genes through several mechanisms (Table 1). For example, two KLF4 binding sites exist in the SM22α promoter region, including an expansively positioned CArG box within its enhancer sequence [35]. Serum response factor (SRF) and myocardin bind to the CArG sites to activate transcription of VSMC contractile genes [36]. KLF4 inhibits SRF and myocardin binding to the CArG site, preventing contractile gene expression [37]. KLF4 also inhibits contractile gene expression through posttranslational histone modifications. KLF4 recruits histone H4 deacetylase activity to VSMC genes, preventing SRF binding with methylated histones and the CArG box [37]. The deacetylation of histone H4 was linked to loss of SRF binding during suppression of VSMC differentiation [37]. Together, these mechanisms illustrate KLF4’s multiple ways of regulating VSMC phenotype switching through transcriptional and epigenetic modifications. By suppressing contractile protein expression, these modifications directly impact VSMC phenotype and aortic wall integrity.

**Table 1:** Molecular influences on vascular smooth muscle cell phenotype and their roles in abdominal aortic aneurysm development (Original table created by authors for current review article)

Regulator	Type	Mechanism of Action	Effect on VSMCs	Impact on AAA	Source(s)
KLF4	Transcription factor	Binds promoter, recruits HDACs, suppresses SRF/myocardin activity	Inhibits contractile gene expression, promotes synthetic phenotype	Stimulates VSMC phenotypic switching, ECM degradation	[18, 35–37]
Myocardin	Transcription coactivator	Activates CArG box-containing promoters via SRF	Promotes contractile marker expression and VSMC stability	Protective against dedifferentiation	[35, 37]
TFEB	Transcription factor	Upregulates lysosomal and autophagy genes	Reduces apoptosis, promotes VSMC survival	Reduces aneurysm formation; loss worsens outcomes	[18]
BAF60c	Chromatin remodeling protein	Permits access to contractile gene promoters by nucleosome repositioning	Maintains contractile phenotype	Downregulated in AAA; loss leads to VSMC dysfunction and increased AAA	[38]

In contrast to KLF4’s suppressive role, TFEB promotes a protective VSMC phenotype (Table 1). As master regulator of lysosomal biogenesis and autophagy, TFEB activation reduces aneurysm formation by restoring lysosomal function and enhancing autophagy in VSMCs [18]. TFEB KO in the Ang-II and elastase-induced AAA model increased VSMC apoptosis and accelerated aneurysm formation [18]. Transcriptomic analysis revealed that TFEB suppresses apoptotic signaling by upregulating anti-apoptotic B-cell lymphoma 2 (BCL2), a key downstream effector [18]. Pharmacological activation of TFEB using 2-hydroxypropyl-ß-cyclodextrin attenuated aneurysm growth in a TFEB-dependent manner [18]. These findings demonstrate that TFEB preserves VSMCs by regulating apoptotic signaling and may serve as a promising therapeutic target to limit AAA progression.

Chromatin remodeling complexes also influence VSMC phenotype. BAF60c is a protein subunit of the SWItch/sucrose nonfermentable (SWI/SNF) chromatin-remodeling complex that interacts with SRF and myocardin to transcriptionally activate VSMC contractile genes [38]. By repositioning or restructuring nucleosomes, chromatin-remodeling complexes like SWI/SNF enable or restrict the expression of specific genes, thus playing a key role in regulating phenotype and function (Table 1). Expression of BAF60c is reduced in human and murine AAA, but the mechanism was previously unclear [38]. Using Angiotensin-II (Ang-II) and elastase-induced AAA models, BAF60c knockout (KO) mice exhibited increased AAA incidence, larger maximum aortic diameters, greater elastin degradation, and enhanced leukocyte infiltration in the aortic wall [38]. These findings indicate that BAF60c plays a protective role in AAA progression by maintaining the VSMC contractile phenotype through chromatin remodeling and promoting the expression of contractile proteins that preserve aortic wall integrity. Targeting chromatin remodeling pathways or specifically restoring BAF60c function may represent a novel therapeutic approach to stabilize VSMC phenotype and slow aneurysmal growth.

The embryonic origin of VSMCs further contributes to this dysfunction. VSMCs in different regions of the aorta arise from multiple embryonic lineages, including neural crest, splanchnic mesoderm, lateral plate mesoderm, and somatic mesoderm [39]. The infrarenal aorta, the most common site of AAA, consists mainly of mesodermal VSMCs, which notably exhibit heightened sensitivity to pro-inflammatory cytokines (e.g., IL-1ß) that induce MMP(2 and 9) expression and ECM degradation [40, 41]. This increased sensitivity promotes local inflammation and ECM degradation, contributing to the infrarenal aorta’s vulnerability to aneurysm formation.

### Apoptosis and Senescence in AAA Progression

Various pathologic triggers also promote VSMC apoptosis, another mechanism responsible for AAA formation [11, 18, 28]. Increased VSMC apoptosis occurs in humans with AAA, as it is indicated as an early indicator of AAA in various historical studies [42, 43]. Apoptotic loss of VSMCs reduces the cellularity required for ECM maintenance and repair, structurally compromising the vessel wall and making it more prone to dilation and rupture. As VSMCs are the primary producers of structural proteins like elastin and collagen, their depletion leads to impaired ECM synthesis, diminished tensile strength, and a loss of wall integrity. Apoptosis also contributes to the release of DAMPs, which exacerbate inflammation and ECM degradation. Various pathways have been identified in VSMC apoptosis. Abnormal TNF-α-endoplasmic reticulum (ER) stress has surfaced as a key mechanism that induces VSMC apoptosis [44]. Specifically, the ER stress pathway Protein Kinase R-like ER Kinase (PERK) was identified via single-cell RNA-Seq of human AAA tissue as a driver of an apoptotic cellular response [44]. PERK activates the integrated stress response via phosphorylation of eukaryotic initiation factor 2 alpha (eIF2α), reducing cell-wide translation [45]. This phosphorylation selectively enhances translation of activating transcription factor 4 (ATF4) [45]. ATF4 then translocates to the nucleus and induces expression of pro-apoptotic genes like C/EBP homologous protein (CHOP), triggering mitochondrial dysfunction, caspase activation, and apoptotic cell death [45]. Both VSMC-specific genetic depletion (*Eif2ak3*^*fl/fl*^* Myh11-CreER*^*T2*^) and pharmacological inhibition in an elastase and Ang-II-induced AAA model sustained VSMC function, reduced elastin fragmentation, tempered VSMC apoptosis, and limited AAA growth [44]. This highlights that targeting the PERK pathway through genetic or pharmacological means can effectively preserve VSMC integrity and prevent key pathological changes that induce AAAs.

Senescence of VSMCs is another important contributor to AAA pathogenesis, as senescent VSMCs enter G1 cell cycle arrest and cease to proliferate. Although dormant, senescent VSMCs exhibit sustained metabolic function and can induce an inflammatory response by secreting cytokines, proteases, and growth factors known as the senescence-associated secretory phenotype (SASP) [46]. This phenotype contributes to chronic inflammation and ECM degradation within the aortic wall, allowing potential aneurysm formation. Senescent VSMCs also secrete large amounts of MMPs for ECM, specifically elastin degradation, which triggers additional inflammation [20]. Also, senescent VSMCs exhibit reduced contractility and an increase in synthetic markers, linking them to the adverse phenotype [41, 47]. Targeting senescent VSMCs or modulating SASP factors may represent a novel therapeutic strategy to mitigate AAA development. While intrinsic stressors like inflammation and oxidative stress drive dysfunctional changes in VSMCs, systemic factors such as sex and gut microbial composition modulate the intensity and persistence of these responses. Both the hormonal environment and microbial-derived metabolites influence the inflammatory and metabolic tone of the vessel wall, shaping how VSMCs respond to stress. From this view, sex and microbiome converge on shared regulatory pathways that determine whether VSMC plasticity remains adaptive and advantageous or pathologic and vulnerable. Recognizing that maladaptive VSMC responses are not solely driven by acute damage but are additionally influenced by systemic physiological inputs provides a more expansive framework for understanding individual variability in AAA risk and progression.

### Gender Differences, VSMC Behavior, and AAA Pathogenesis

Sex dimorphism in AAA is characterized by incidence, growth rate, rupture susceptibility, and surgical outcomes. While men are at a higher risk for developing AAA, women tend to present with more advanced disease, higher rupture rates at smaller sizes, and worse postoperative survival [5, 7]. Various sex-related factors have been suspected to be influential, such as differences in hormonal profiles, inflammatory signaling, and VSMC behavior, suggesting that sex hormones and sex-linked gene regulation influence VSMC phenotypes in distinct ways [3]. Estrogen is suspected to exert protective effects on the aortic wall by promoting VSMC contractility, reducing MMP expression, and enhancing NO bioavailability [48, 49]. However, one review reported men with AAAs had higher circulating estradiol and progesterone levels compared to healthy controls [48]. This study admittedly however, had key limitations, including the inability to collect blood samples longitudinally. In contrast, when supplemented with estradiol, male mice displayed decreased MMP levels within aortic tissue, similar to the baseline female MMP levels [50]. These contradictory findings reveal the complexity of sex hormone signaling in cardiovascular health and AAA, and point to the need for longitudinal, mechanistic studies to clarify associations.

Estrogen interacts with three main receptors: estrogen receptor α (ER-α), G protein-coupled estrogen receptor (GCER), and estrogen receptor β (ER-β), with ER-α being the main receptor [51]. ER-α is highly expressed in VSMCs, and its importance is highlighted by the loss of estrogen’s protective effects against vascular injury in ER-α gene knockout (KO) mice [52, 53]. Estrogen also prompts VSMCs to enter G1 cell cycle arrest, acting as a roadblock to further proliferation seen in the synthetic VSMC phenotype [54]. In the elastase perfusion model, male rats showed decreased levels of MMP-9 and MMP-2 and slower AAA progression than control male rats when administered exogenous estrogen, indicating the protective role of estrogen in preventing ECM and vessel wall degradation [55]. Estrogen also prevented phenotype switching of VSMCs in an aortic dissection mouse model [51]. Although estrogen can upregulate KLF4 expression in vascular tissues, this introduces a complex dynamic [56]. Since KLF4 is a known promoter of the synthetic phenotype, its upregulation by estrogen would seem contradictory to estrogen’s protective role in AAA. This paradox suggests that estrogen’s effects may depend on cellular context, receptor subtype signaling, and that its protective mechanisms likely involve additional or overriding pathways beyond KLF4 regulation alone. Overall, these findings show how estrogen and its receptors help preserve VSMC homeostasis by limiting phenotype switching, suppressing MMP activity, and maintaining ECM integrity. Despite some conflicting data regarding mechanistic control of KLF4, estrogen’s overall influence appears to favor stabilization of the contractile phenotype and protection against excessive vascular remodeling. This multifaceted regulatory role may help explain the lower incidence of AAA in premenopausal women, while also demonstrating how the loss of estrogenic signaling post-menopause may contribute to increased aneurysm progression and rupture risk [7].

Androgen activity also may play a role in AAA formation. In studies on testosterone’s influence on inflammation, testosterone deficiency was correlated with an increased level of inflammatory markers, and long-term testosterone therapy helped mitigate inflammation in hypogonadal men [57]. Testosterone was also found to be decreased in male AAA patients [58]. These results provide valuable insight into AAA progression, as chronic inflammation plays a critical role in VSMC dysfunction and aortic wall degradation, suggesting that testosterone deficiency could be a contributing risk factor for aneurysm development through pro-inflammatory mechanisms. Testosterone deficiency is correlated with aging in males, independent of comorbidities such as hypertension or dyslipidemia [59]. This association could account for the higher incidence of AAA in men over the age of 65 but will require further research to determine the possibility of a causal relationship.

There are also sex-based differences in VSMCs. In males, VSMCs exhibit elevated cytosolic Ca^2+^ concentrations due to several mechanisms, including upregulation of the STIM/Orai1 pathway in the sarcoplasmic reticulum (SR), increased Ca^2+^ influx through calcium channels driven by testosterone-induced protein kinase C delta (PKCδ) activation, and enhanced membrane depolarization via greater Na^+^-K^+^−2Cl^−^ cotransporter (NKCC) [60]. Elevated cytosolic Ca^2+^ levels enhance myosin light chain phosphorylation and actin-myosin cross-bridge cycling, which increases VSMC contractility and stiffness under normal physiological conditions [61]. Under stress, such as inflammation or acute injury, this high calcium environment can shift from being versatile to dysfunctional. Sustained calcium elevation activates downstream signaling pathways such as calmodulin-dependent kinases (CaMKs), which drive gene expression patterns associated with pro-synthetic and pro-inflammatory VSMC phenotypes [62, 63]. Conversely, female VSMCs exhibit lower baseline cytosolic Ca^2+^ concentrations than males, partly due to differences in the expression and behavior of Ca^2+^ signaling molecules like stromal interaction molecule 1 (STIM1) [60]. STIM1 senses ER calcium depletion, which activates store-operated calcium entry (SOCE) through Orai channels. VSMC stiffness is regulated by intracellular calcium through myosin-driven contraction and calcium-sensitive adhesion pathways [32]. Lower cytosolic Ca^2+^ levels in females likely support the contractile VSMC phenotype, whereas higher Ca^2+^ in male cells predisposes cells to the synthetic phenotype [60]. High calcium levels are linked to an osteogenic VSMC phenotype that leads to arterial calcification and stiffening, increasing AAA susceptibility [20]. Progesterone also induces relaxation of vascular smooth muscle, decreasing blood pressure and contributing to the reduced risk observed in premenopausal women [64]. However, this protection is reduced post- menopause as protective female hormone levels decline and CVD risk increases [65]. Future research should consider sex-specific Ca^2+^signaling pathways in VSMCs and potentially utilize hormonal modulation of calcium-handling proteins as therapeutic targets. These strategies, as well as further research, may help close critical gaps in screening, diagnosis, and treatment strategies for AAA, especially between genders [66].

### Gut Dysbiosis and the Gut-Vascular Axis in AAA

**Fig. 1:**
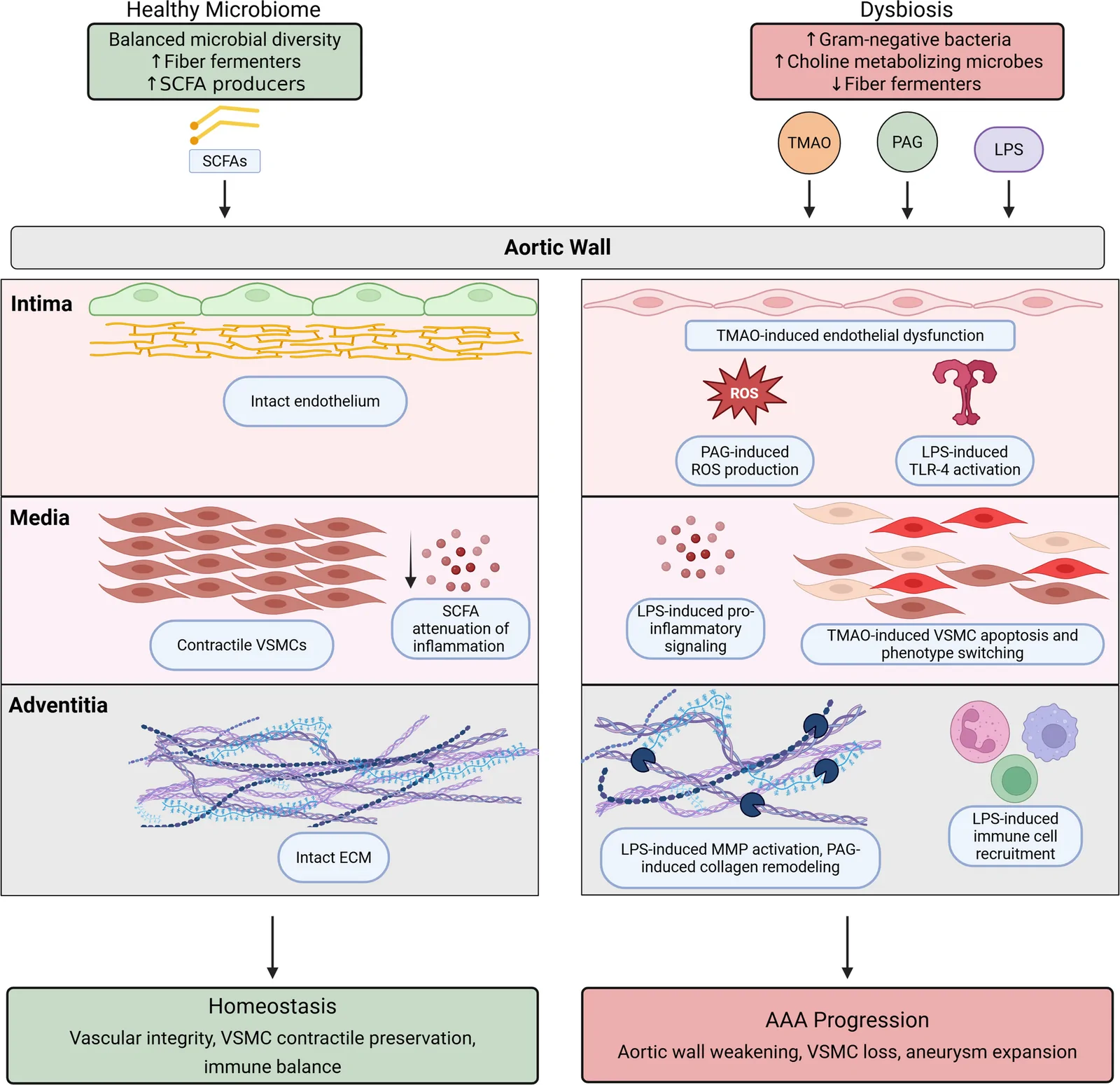
Effects of Gut Dysbiosis on resident aortic tissues. A healthy microbiome (left) is characterized by balanced microbial diversity and elevated short-chain fatty acid (SCFA) producers promotes vascular homeostasis, which includes vascular smooth muscle cell (VSMC) contractile phenotype preservation and immune balance. SCFAs attenuate inflammation in the media, presenting a potential protective mechanism. Dysbiosis (right) drives the production and circulation of the pro-inflammatory metabolites trimethylamine oxide (TMAO), phenylacetylglutamine (PAG), and lipopolysaccharide (LPS). These metabolites exert layer-specific effects within the aortic wall. Within the intima, TMAO induces endothelial dysfunction, PAG increases ROS production, and LPS activates toll-like receptor 4 (TLR-4) signaling. In the media, TMAO promotes VSMC apoptosis and phenotypic switching, while LPS induces pro-inflammatory signaling of cytokines. In the adventitia, LPS induces immune cell recruitment and matrix metalloproteinase activation, while PAG induces collagen remodeling, collectively driving AAA progression. Original figure created by authors using BioRender

The gut microbiome is a regulator of both cardiovascular and metabolic health, with growing evidence linking gut microbial composition to outcomes in both systems [67]. Typically, three phyla make up the human gut microbiome: *Bacteroidetes* (9%−42%), *Firmicutes* (30%−52%), and *Actinobacteria* (1%−13%) [68]. Disturbances in the proportions of these bacteria are deemed “dysbiosis” and can contribute to various morbidities, including cardiovascular [69]. Gut microbiota is necessary for digestion and certain nutrient absorption (e.g., complex polysaccharides) when confined to its proper location and in its balanced composition. Various endogenous and exogenous risk factors can contribute to gut dysbiosis, altering the composition of the gut microbiome. Pollution, antibiotics, poor diet (e.g., low fiber intake, high cholesterol intake), and systemic inflammation are all risk factors for gut dysbiosis [70]. Once modified, the gut microbiome can have varying effects on both cardiovascular and metabolic health [71]. These disruptions can allow gut bacteria to translocate and spread systemically, a phenomenon observed in conditions like inflammatory bowel diseases (IBD), sepsis, and urinary tract infections (UTIs) [72]. Other examples of transmission of bacteria causing infection include blood-borne transmission and aortic aneurysm surgery [73]. Rectal colonization by *Streptococcus pyogenes* led to infection of the abdominal aortic wall, subsequently inducing AAA formation [74]. A key factor in this process is an increase in pathogenic bacteria (i.e., *Staphylococcus*, *Streptococcus*, and *Enterobacteriaceae*), which can cause epithelial damage in the gastrointestinal tract and promote inflammation. Epithelial damage can compromise gut barrier integrity, allowing bacteria and their metabolic products to enter the circulation and trigger further inflammation and vascular injury [75]. Systemic inflammation and vascular injury both prompt VSMCs to switch to the synthetic phenotype [76]. While this phenotype supports vascular remodeling following injury, persistent inflammation driven by gut dysbiosis sustains the synthetic state, ultimately impairing vascular stability and accelerating aneurysm formation. Production of microbial metabolites can also play a role in triggering an inflammatory response. Metabolites such as TMAO, PAG, short-chain fatty acids (SCFAs), and lipopolysaccharides (LPS) have been associated with CVD risk through adverse interactions with their receptors [17] (Fig. 1).

### TMAO

TMAO was one of the first metabolites to be studied as a potential link between the gut microbiome and CVD, with subsequent studies linking it to metabolic disorders, including insulin resistance and chronic kidney disease (Table 2) [77]. TMAO is originally derived from phosphatidylcholine, which is found in red meat. The gut microbiome metabolizes phosphatidylcholine into choline and the TMAO precursor, trimethylamine (TMA) [78]. TMA is then transported to the liver via the portal vein, where it is oxidized by flavin-containing monooxygenase 3 (FMO3) into TMAO [79]. TMAO is associated withimpaired cholesterol clearance, enhanced platelet reactivity, increased foam cell formation, pro-inflammatory signaling, and reduced EC function [80]. More recently, TMAO is implicated in promoting VSMC phenotypic switching toward the synthetic phenotype [81]. Elevated TMAO levels activate the NF-κB pathway, which upregulates pro-inflammatory cytokines and increases expression of synthetic markers in VSMCs while downregulating contractile markers [81]. In humans, patients with AAA had significantly elevated plasma TMAO levels, with a higher likelihood of AAA in men but not women [17]. This suggests that the mechanism of action of TMAO may differ between sexes but requires further research to define an exact relationship. Mice were fed a high-choline diet to encourage TMAO production, undergoing the Ang-II or topical porcine pancreatic elastase-induced AAA model [17]. Inhibition of gut microbiota via broad-spectrum antibiotics limited AAA growth and downregulated the PERK stress response [17]. Additionally, TMAO increased maximal aortic diameter, AAA incidence, extent of elastin degradation, and accumulation of senescence markers p21 and p16, as well as MMP-2 in an Ang-II mouse model [82]. This links TMAO to many of the underlying processes that increase AAA susceptibility, as ROS affect cell growth, immune response, and aging [83]. Fluoromethylcholine, a TMAO inhibitor, has also been observed to slow aneurysm progression via anti-inflammatory, antioxidant, and anti-fibrotic properties [79]. Although there are strong associations between TMAO and AAA, as well as other CVDs such as atherosclerosis and heart failure, the exact mechanisms of action of TMAO remain an active area of investigation [84].

**Table 2:** Gut-derived microbial metabolites and their proposed mechanisms of vascular injury, with effects on VSMCs and potential sex-specific influences in AAA pathogenesis (Original table created by authors for current review article)

Metabolite	Source Derived	Primary Mechanism(s) of Vascular Injury	Impact on VSMCs	Sex-specific Effects	Source(s)
Trimethylamine oxide (TMAO)	Gut metabolism of choline via *FMO3*	Promotes inflammation, endothelial dysfunction, foam cell formation	Induces phenotypic switching and apoptosis	Elevated levels in men with AAA	[17, 77, 79–81, 83]
Phenylacetylglutamine (PAG)	Phenylalanine metabolism + liver metabolism	Increases platelet aggregation, ROS, systemic inflammation	Enhances stress signaling and thrombosis	Not yet clearly defined	[86–88]
Lipopolysaccharide (LPS)	Cell wall component of gram-negative bacteria	Activates TLR-4, systemic inflammation	Triggers pro-inflammatory signaling in VSMCs	Differential TLR-4 responses across sexes	[91–93]
Short-chain fatty acids (SCFAs)	Fermentation of dietary fiber	Immune modulation (i.e., Treg promotion, cytokine regulation)	May attenuate inflammationlimited AAA data	Potential immunoregulatory benefits in females	[94–98]

### PAG

Phenylacetylglutamine (PAG) is derived from the essential amino acid phenylalanine and is linked to AAA and CVD. Originally a marker for obesity, elevated atherosclerosis risk, and type II diabetes, PAG is now considered a marker of adverse cardiovascular outcomes [85]. Gut microbiota convert phenylalanine into phenylacetic acid, which is metabolized by the liver into PAG [86]. Researchers found a positive correlation with heart failure severity and the level of circulating PAG [87]. Recent research has explored the relationship between PAG and the inflammatory response and found that PAG increases ROS and induces systemic inflammation through pro-inflammatory cytokines [88]. This heightened oxidative and inflammatory environment drives VSMCs to adopt the synthetic phenotype commonly seen in AAA. Additionally, PAG increased platelet responsiveness and thrombosis potential by interacting with G-coupled adrenergic receptors, such as ADRB2, ADRA2A, and ADRA2B [77, 88]. Enhanced platelet activation and thrombus formation exacerbate vessel wall stress and local hypoxia, further promoting VSMC dysfunction and localized inflammation, creating an environment vulnerable to aneurysm formation and progression [89]. Taken together, PAG represents an important microbial metabolite capable of contributing to AAA pathogenesis through its combined effects on inflammation, oxidative stress, and thrombus-mediated vascular remodeling.

### LPS and TLRs

Toll-like receptors (TLRs) are innate sensors that play a key role in mediating inflammation and immune activation in various tissues [90]. In the context of AAA, TLR-2 and TLR-4 are of interest due to their expression on VSMCs and their ability to respond to gut-derived microbial signals (Table 2). One such signal is LPS, an endotoxin derived from gram-negative bacterial cell walls, which increases systemically during gut dysbiosis [91]. Activation of TLR-4 by LPS initiates NF-κB signaling within VSMCs, upregulating pro-inflammatory cytokines, MMPs, and ROS [92]. In contrast, TLR-2 may exert a more protective function. TLR-2 activation can attenuate aneurysm formation, while TLR-2 KO mice exhibit increased AAA formation in the Ang-II-induced model [93]. These findings illustrate how dysregulated TLR signaling within VSMCs, potentially initiated by gut microbial imbalances, can contribute to VSMC dysfunction. With this, the TLR-VSMC axis serves as a mechanistic link between gut dysbiosis and aortic wall weakening and may offer novel targets to modulate inflammation and preserve VSMC stability in AAAs.

### SCFAs

Short-chain fatty acids (SCFAs) are also influential in AAA development (Table 2). SCFAs, namely formate, acetate, propionate, and butyrate, are the products of fermentation from gut microbiota. SCFAs are derived from non-digestible carbohydrates that become accessible to gut microbiota following digestion [94]. These products can affect various processes throughout the body, such as inflammation, glucose, and lipid metabolism [94, 95]. With inflammation, SCFAs act as a mediator between the gut microbiome and immune cells. SCFAs contribute to intestinal immune homeostasis by modulating T-cell polarization and promoting T-cell differentiation into both effector and regulatory T cells (Tregs) [96]. Additionally, SCFAs influence neutrophil activity by exerting both stimulatory and inhibitory effects, and they also regulate monocyte and macrophage activity [97]. SCFAs further affect inflammation by controlling cytokine production in immune cells, shaping the overall inflammatory response, and contributing to immune tolerance or activation [98]. Various animal studies have provided compelling evidence that modulation of the gut microbiome influences AAA progression. In male Apolipoprotein E-deficient mice, oral supplementation of antibiotics was shown to attenuate aneurysmal dilation and reduce the risk of rupture [99]. Suppression of the gut microbiome via antibiotics limits the production of previously discussed metabolites and highlights the contributory role of these metabolites in propagating vascular inflammation and aneurysm development [100].

## Conclusion

Sex differences and gut dysbiosis correlate with AAA pathogenesis. This review examined how each can exert effects on VSMCs and specifically the phenotypic change observed in AAA. Estrogen exerts protective effects in healthy phenotypes and tissues, while testosterone is pro-inflammatory and pro-apoptotic. In parallel, gut dysbiosis allows metabolites to leak into circulation, interacting with immune cells and VSMCs, inducing inflammatory responses, and leading to ECM degradation. Despite associative evidence between these factors and AAA pathogenesis, mechanistic details are needed. Further insights into the gut microbiome-gender-AAA axis may reveal novel biomarkers and therapeutic targets to slow AAA growth and improve outcomes, which is especially important in an aging population.

## Key References

- Golledge J, Thanigaimani S, Powell JT, Tsao PS. Pathogenesis and management of abdominal aortic aneurysm. European Heart Journal. 2023;44:2682–97.
  - ○ Review article outlining current management and therapies for abdominal aortic aneurysms.
- Qian G, Adeyanju O, Olajuyin A, Guo X. Abdominal Aortic Aneurysm Formation with a Focus on Vascular Smooth Muscle Cells. Life. 2022;12:191.
  - ○ Review article highlighting how vascular smooth muscle cell dysfunction contributes to abdominal aortic aneurysms.
- Cao, G., Xuan, X., Hu, J. et al*.* How vascular smooth muscle cell phenotype switching contributes to vascular disease. Cell Commun Signal 20, 180(2022).
  - ○ Review article examining the influence of vascular smooth muscle cell phenotype switching on various vascular diseases.

## Data Availability

No datasets were generated or analysed during the current study.

## Abbreviations

AAs: Aortic aneurysms
AAA: Abdominal aortic aneurysm
Ang-II: Angiotensin II
ATF4: Activating transcription factor 4
BCL2: B-cell lymphoma 2
CaMKs: Calmodulin-dependent kinases
CHOP: C/EBP homologous protein
ECM: Extracellular matrix
eIF2α: Eukaryotic initiation factor 2 alpha
ER: Endoplasmic reticulum
ER-α: Estrogen receptor α
ER-β: Estrogen receptor β
FMO3: Flavin-containing monooxygenase 3
GCER: G protein-coupled estrogen receptor
IBD: Inflammatory bowel diseases
IL-6: Interleukin 6
IL-1α: Interleukin-1 alpha
IL-1β: Interleukin-1 beta
IL-1: Interleukin-1
IFNγ: Interferon gamma
KLF4: Kruppel-Like factor 4
LPS: Lipopolysaccharides
MCP1: Monocyte chemoattractant protein 1
MMP2: Matrix metalloproteinase 2
MMP9: Matrix metalloproteinase 9
MYH11: Smooth muscle cell myosin heavy chain
NKCC: Na^+^-K^+^-2Cl^−^ cotransporter
NO: Nitric oxide
PAG: Phenylacetyl glutamine
PERK: Protein Kinase R-like ER Kinase
PKCδ: Protein kinase C delta
RNA-Seq: RNA-Sequencing
SASP: Senescence-associated secretory phenotype (SASP)
SCFAs: Short-chain fatty acids
SMα: Smooth muscle alpha actin
SM22α: Transgelin
SRF: Serum Response Factor
SR: Sarcoplasmic reticulum
STIM1: Stromal interaction molecule 1
SWI/SNF: SWItch/sucrose nonfermentable
TFEB: Transcription factor EB
TLR: Toll-like receptors
TMA: Trimethylamine
TMAO: Trimethylamine oxide
TNFα: Tumor necrosis factor alpha
UTIs: Urinary tract infections
VSMC: Vascular smooth muscle cell
